# Medical Association of Georgia

**Published:** 1899-05

**Authors:** 


### SOCIETY REPORTS.

### MEDICAL ASSOCIATION OF GEORGIA.

*Abstract of the Proceedings of the Fiftieth (Semi-Centennial) Annual Meeting, held at Macon, Ga., April 19, 20, and 21, 1899.*

### FIRST DAY—MORNING SESSION.

The Association convened in the Academy of Music, and was called to order at 10 A.M. by the President, Dr. Howard J. Williams of Macon.

Prayer was offered by the Rev. J. W. Roberts, D.D., of Macon.

Hon. Emory Speer, Judge of the United States Court, Macon, was introduced by the President, and delivered an eloquent address of welcome.

In the absence of Dr. DeSaussure Ford of Augusta, at the invitation of the President, Dr. Virgil O. Hardon of Atlanta, responded to the Address of Welcome in a graceful and befitting manner.

At the close of Dr. Hardon's remarks, Dr. Williams delivered his address as President of the Association. He selected as his subject "The Surviving Members of the First Meeting of the Medical Association of Georgia." The address was in the form of a poem.

Dr. H. McHatton made a short report on behalf of the Committee of Arrangements in which he outlined the social features and entertainments for the Association and its guests.

After the transaction of routine business, the reading of papers was proceeded with.

Dr. T. Virgil Hubbard of Atlanta, read a paper entitled "The Eliminative and Antiseptic Treatment of Typhoid Fever."

While there may be some room for justly criticizing the size of dose and frequency of administration, advocated by Dr. Woodbridge, there can be no doubt that to him belongs the honor of having combined a number of drugs for the treatment of typhoid fever, which, if systematically and continuously used, will reduce the mortality of this disease. While the author has used the same drugs, but in larger doses, and not so frequently administered, he is not prepared to say that his method of administration is not the better, for he has never used it that way, and he is not willing to criticize his (the Woodbridge) plan before having tried it. The most conspicuous and pronounced opponents of the antiseptic and eliminatve treatment of typhoid fever are almost, without exception those men, who have never systematically used it in even a limited number of cases, thus placing themselves in the absurd position of condemning this treatment without ever having given it a fair trial. A number of physicians will testify to the remarkable tolerance of the system to mercury, when under the influence of the poison of diphtheria, some having given as high as 300 grains of calomel in one week to an adult with diphtheria without producing salivation or violent purgation. He would not attempt to offer any explanation of why this is so, but the fact remains. He has given a half grain of calomel to a typhoid patient every two hours for eighty-four hours without producing salivation or purgation. While the same tolerance does not exist in typhoid fever that syphilis and diphtheria furnish, yet it does not seem unreasonable to assume that there is some physiological antagonism between the disease and mercury, which may account to a certain extent for its controlling influence. The treatment the author has used, as suggested to him by Dr. Stockard of Atlanta, consists in the administration of the following: Calomel, $\frac{1}{2}$ grain; guaiacol carbonate, 2 grains; podophyllin, $\frac{1}{20}$ grain. Given in one capsule every two hours for twenty-four to forty-eight hours, depending on the condition of the bowels. He continues this until he has secured four or five intestinal evacuations for two successive days, and then he leaves off the calomel and adds half grain of menthol to the guaiacol and podophyllin. If, after discontinuing the calomel, there is any tendency of the bowels to become inactive, he administers a small dose of salts or Hunyadi water in the morning. He always endeavors to secure at least two or more evacuations daily, depending on the temperature and tympanites. If, after four or five days of treatment, the temperature remains high or rises after having remained stationary, he again resorts to the calomel as above, for twenty-four hours, and it invariably reduces the temperature and results in a general improvement in the patient's condition. He continues the administration of the guaiacol, menthol and podophyllin throughout the disease.

Regarding the variation in size of dose and frequency of administration of the calomel, the common sense and good judgment of the physician must be relied upon for the successful administration of this treatment. Timidity or skepticism on the part of a physician will too often result in failure, but the continuous and apparently heroic administration in the beginning of treatment is the *sine qua non*, and will invariably be rewarded by a favorable modification of the course and symptoms of typhoid fever.

With reference to diet and the general hygiene of the sick-room, typhoid patients can retain, digest and assimilate a larger amount of nourishing food with this treatment than with any other. The author is in favor of giving more food to typhoid patients than has been the custom heretofore under the old régime.

Dr. Gillham P. Robinson, of Atlanta, followed with a paper entitled "Infant Feeding in Health and Disease."

The proper suggestions for infant feeding and the rules governing substitute food should emanate from the medical profession rather than from the non-medical proprietors of commercial foods. The position occupied by the physician in comparison with that of the venders of patent foods is a very humiliating one and should no longer exist. He would not condemn the use of all commercial foods, as some of them were useful for children who have passed their first years, but what he condemns is the pernicious use of these foods by the profession simply because they may have agreed with some individual children, or because some old nurse advises it. The commercial foods are far from being reliable, and all of them, even when containing the percentages of fats and proteids claimed for them by their owners, may have these constituents reduced below the limits of nutrition when properly diluted for infant feeding. The essayist endeavored to make clear how cow's milk can be so modified as to closely resemble mother's milk. Physicians should refuse to be dictated to by proprietors of so-called infant foods, or by old nurses. They should think for themselves, prepare the food scientifically, be on the watch for improvement, and, above all, hold self-respect. Dr. Robinson has no fear of the result when modified milk is used with care and accuracy.

Dr. R. M. Harbin, of Rome, read a paper in which he reported "Seven Cases of Diphtheritic Croup, Two Aborted by Antitoxin, and Five Cured by Antitoxin and Intubation."

Dr. Harbin drew the following conclusions:

1. Numerous statistics prove that antitoxin has lessened the mortality of diphtheria one-half.

2. The remedy is never toxic in its effects, and never causes or increases albuminuria, if properly used, and does not interfere with the use of other remedies.

3. The dose of antitoxin in laryngeal cases should be from 1500 to 4000 units, according to the age and the condition of the patient.

4. In weak, anemic and albuminuric cases, its administration should be more guarded, as the restraining effect on the kidneys, as in one of his cases, may be due to a faulty administration.

5. Antitoxin favors resolution of the membranes in cases subjected to intubation, lessening the absorption of toxins and rendering an early removal of the tube safe.

6. Antitoxin has a favorable effect on the mixed infections.

7. From a personal observation of three successful out of five tracheotomies and five consecutive successful intubations for croup, his preference is for intubation, except in rare cases.

FIRST DAY—AFTERNOON SESSION.

Dr. O. B. Bush, of Colquitt, read a paper on "The Necessity of a State Pediatric Society."

The author advanced several reasons why such a society should be organized, and said there is a large number of physicians in the country, as well as in cities, who do not know what disease they are treating when they are called to see a child, on account of the obscurity of the symptoms. The consequence is, the mortality among these little sufferers is appalling sometimes.

"The Endoscopic Treatment of Chronic Urethritis." Dr. W. L.

Champion, of Atlanta, read a paper on this subject in which he presented a few facts relative to the treatment of this disease which he hoped would result in the more general use of the endoscope. A careful study of the lesions of chronic urethritis, through the endoscope, will convince the most skeptical of the value of direct local treatment in these conditions. From practical experience the author states that a large percentage of inflamed urethras remain uncured for months, and frequently years, when the proper endoscopic treatment would perfect a speedy cure. After several years' use of the endoscope in treating these conditions it has been his good fortune to relieve many suffering individuals, if not from pain, from the mental anxiety produced on account of a chronic urethral discharge, or the possibility of a stricture in the near future. It is only after careful study and some experience with the use of the endoscope that the various pathological conditions can be recognized.

In chronic urethritis, not due to cicatricial stricture, his experience has been that in a majority of cases the seat of inflam. mation is in the middle urethra, or, more properly speaking, the lower portion of the spongy portion. That the spongy portion of the canal is more frequently involved than the membraneous or prostatic there can be no doubt, but he is convinced that in every case of chronic urethritis the posterior urethra is more or less involved. This can be demonstrated by taking a patient with a few ounces of urine in his bladder, whose urethra is inflamed and bladder is not involved, and irrigate the anterior urethra thoroughly, and then have the patient pass his urine in a glass, and invariably shreds of filaments will be noticed. The endoscope should not be used when the urethra is acutely inflamed, or so long as the urine appears cloudy from an abundance of mucus. The endoscope which he believes to be one of the best is the W. K. Otis with Klotz's endoscopic tubes.

The author could report quite a number of cases of chronic urethritis that had extended over a period of from one to fifteen years that have been permanently cured by the use of the endoscope, but he did not think it wise to bore the Association with their histories. He stated, however, that many of these cases had been intelligently treated, the stricture, if one had been present, was properly dilated or cut, the meatus was incised, the urethra injected or irrigated, but the chronic inflammatory condition remained, which was promptly relieved by proper endoscopic treatment. The number of cases that respond to endoscopic treatment after other means have failed certainly demonstrates the value of the endoscope.

Dr. H. A. Hare, of Philadelphia, contributed an address (by invitation) entitled "Some Facts in Regard to the Medical Complications, Sequelæ and Treatment of Typhoid Fever," which was read by Dr. Hurt in the absence of the author. (To be published in this JOURNAL.)

Dr. Dunbar Roy of Atlanta, read a paper on "Some of the Fallacies in the Modern Treatment of Nose and Throat Diseases."

By fallacies the author means the false recognition of pathological conditions, and the wrong remedy for the condition when it is recognized. As to the use of sprays, they are exceedingly valuable in a large number of cases, but the indiscriminate use of them in every case that consults the physician is sometimes harmful and often injurious. The author has come to find that there is such a thing as the spray habit just as we find the cocain and morphine habits. This is brought about largely by specialists who consider that it is necessary for patients to come to their offices every day or every other day and be sprayed out, until it not only becomes a habit with the patient, but a mercenary habit with the physicain. A spraying out makes the patient feel temporarily better and in one whose nasal chambers have been closed the sensation felt of opening up is delightful, but unfortunately there is a reaction and the opened condition does not last. When he first began to treat diseases of the nose he, too, was an advocate of much spraying, but time has shown him the fallacy of such a procedure. There are two fallacies connected with the use of nasal sprays, which he has found to exist with undue frequency among rhinologists. The first is, that the pressure used in atomizing the liquids is frequently too strong, and the result is that the mucous membrane is injured through such traumatism, and therefore injury instead of benefit is the result. The mucous membrane in the nose is tender and sensitive, although it is sometimes sorely tried and withstands the onslaughts of many odoriferous particles, and for this reason the force of any spray should be well regulated. Some cases require a stronger spray than others, as for instance, in atrophic rhinitis, where irritation of the mucous membrane is considered by some as the rational therapy. In the second place, it has become almost a universal habit to use some oily product as the base of spray medicaments in contradistinction to watery sprays formerly used, and this, too, done as a habit without proper discrimination. He holds that spraying of the nasal chambers is never justifiable unless there is a catarrhal discharge from the membrane readily recognized on anterior rhinoscopical examination, or where there is an accumulation of mucous coming from the contiguous cavaties, such as the frontal or ethmoidal sinus or the maxillary antrum.

The electrocautery is an exceedingly useful instrument in the domain of rhinology and laryngology, but like sprays it has been abused. In his earlier practice he used the electrocautery ten times where he now uses it once. Its great service in the nose is for the reduction of true hypertrophy of the membrane covering the turbinates, when this latter is pouring off an unhealthy discharge, or is so blocking the lumen of the nasal cavities as to prevent the passage of air thoroughly through the same.

Another error frequently committed is the removal of cartilaginous spurs and exostoses or bony spurs from the nasal septum. With more ripened experience he finds that fewer spurs should be removed from the septum, and only in those cases where other well recognized methods of treatment have been used and failed. His reasons for this are that after a spur has been removed from the nasal septum one is obliged to have scar tissue left as the result, and this will always give the patient a point where mucous and scabs will gather to the constant annoyance of the patient, proving sometimes more annoying than the slight obstruction itself. Close discrimination and the knowledge of when to operate should be the aim of every physician, be he specialist or not.

There are fallacies in medicine of two kinds: First, that which is the result of ignorance from the lack of previous training and study; second, that which comes from making a snap diagnosis without studying minutely the case. Both classes are guilty, and in the long run the beneficent public will find out the physician upon whom they may depend.

Dr. M. F. Carson of Griffin, read a paper on "Mitral Stenosis."

When one examines a well-marked case of this disease, he notices on inspection that the apex beat of the heart is in its normal situation, the meaning of this being that the left ventricle is not enlarged, although it may be diminished in size. This depends upon the fact that its work is lessened, owing to the auricle not being able to discharge so much blood into it as it does in health. But this only applies to cases of pure mitral construction. In mitral regurgitation the auricle which is overfilled at great pressure by the blood poured into it through the mitral orifice, discharges its contents again into the left ventricle with accumulated force, as soon as the ventricle ceases to contract, and the latter hypertrophies to meet the abnormal strain. The author next referred to palpation, percussion, and auscultation in this disease.

He divided the course of the disease into two periods. The first, that of good compensation; the second, that of bad failing compensation.

The first and most essential point in the treatment of heart-failure in cases of valvular disease is rest. The patient should be put to bed. This measure of itself sometimes gives excellent results. If venous congestion is very great, bleeding should be resorted to in mitral stenosis. Digitalis still holds the foremost place where the pulse is rapid, feeble, and irregular, and in many cases its effects are very striking. The author believes it is useless to give this drug with a slow pulse, that is, where the pulse is beating at a rate which is normal to the individual in question or below normal. If, we add to the power of each contraction of the heart, we diminish the number of beats per minute, unless, indeed, when we begin to give digitalis the heart beats are abnormally hurried as well as feeble. Under such circumstances digitalis sometimes fails. Strophanthus may then be tried, but the latter drug is far less frequently effectual than digitalis. Digitalis may be combined with strychnine. Another favorite drug with the author, if the urine is scanty, is citrate of caffeine. Abundant and simple food in small quantities, often repeated, is most essential in the successful treatment of this condition.

Dr. John R. Rose of Eastman, read a paper on "The Therapeutic Value of Hypnotism."

The author quoted extensively from the writings of Bernheim, Gerling, Fricke, Beaunis, Lang, Wetterstrand, Kuh, and many others. He said that the following four rules formulated by Bernheim and Beaunis should always guide the physician when undertaking this treatment:

1. Never use hypnotism without the consent of the subject or his legal guardian.

2. Never produce hypnosis except in the presence of a third party standing in some relationship to the subject.

3. Never make suggestions without the patient's consent, except those necessary to effect a cure.

4. Never use authority over a patient to procure this consent if there is reason to expect disagreeable results from the experiment.

To these the author added another one:

5. Carefully weigh words.

Dr. Willis F. Westmoreland, of Atlanta, demonstrated the technique of an operation for varicose veins. The object of the removal of these veins is simply to force the finest blood current through the deeper veins. A number of methods have been suggested for this purpose, which he did not think it was necessary to go over, but the majority of them do not accomplish what the surgeon has in mind. In correcting or removing varicose veins, if the surgeon does not restore the circulation, if the blood current is not sent back through the deeper veins, the condition for which the operation is undertaken is not corrected. The anomalous veins of the the lower extremities are so great and frequent that he knows of no work on anatomy which deals with the anomalies that the surgeon encounters. The operation he proposes has for its object to send the circulation to the deeper femoral veins and the complete removal of the internal saphenous vein. If this vein is taken out completely, circulation through the deeper vessels would be restored. This operation is attended with no more danger than any of the other simpler operations that have been suggested. The length of the incision depends upon the length of the patient's leg. If the surgeon ligates all branches individually it is a tedious procedure, and Dr. Westmoreland showed by colored drawings the method of clamping the individual veins instead of making double ligation.

SECOND DAY—MORNING SESSION.

Dr. H. R. Slack, of La Grange, read a paper on "Catarrhal Laryngitis."

With the hope of alleviating those suffering with this affection, Dr. Slack submits the following prescription, which was suggested to him by Dr. Joseph Holt, of New Orleans, and which he has tried many times with the happiest results:

| | | |
|---|---|---|
| ℞ | Chloralis | 5 grams. |
| | Potasii Bromidi | 3 grams. |
| | Ammonii Bromidi | 2 grams. |
| | Aquæ Cinnamonii | 60 *c. c.* |

*M. Sig.* Teaspoonful, and repeat in twenty minutes, if not relieved.

This is intended for a child about five years old. For younger children he diminishes the dose slightly, although he finds children bear chloral nicely in doses altogether out of proportion to their ages. The chloral relieves the spasm of the larynx, and the bromides allay the nervousness, so that the little patient is soon asleep, and awakes the next morning as well as usual, with little danger of an attack the next night. The first dose generally gives relief, and he has never failed to secure it with the second.

Dr. St. J. B. Graham, of Savannah, read a preliminary report of the discovery of ameba and plasmodia in cultures from the blood of persons suffering from malaria and paludal fevers, and the discovery of motile bodies in human blood plasma and their growth in pure culture, and made some remarks pertaining to the subject.

Dr. W. S. Elkin, of Atlanta, read a paper on "Surgical Treatment of Empyema."

The surgical treatment of empyema will depend upon the condition of the patient, the amount of pus present, and the pathological condition of the lung at the time of operation, and consists of thoracentesis, thoracotomy and thoracoplasty. The objects to be obtained are the removal of the pus and the obliteration of the pus cavity by the safest and most perfect plan. Prompt surgical interference is just as imperative in these diseases as in lumbar, pelvic and liver abscesses, or pus in the peritoneal cavity. Thoracentesis is not in itself a surgical procedure that cures empyema, but a valuable and indispensable means of determining the presence of pus and improving temporarily the condition of the patient before thoracotomy is done.

The author presented a patient that he had operated on at the Grady Hospital about six months ago. He performed Estlander's operation as modified by Schede, and gave a full history of the case as taken from the hospital records. Thirty-eight ounces of thick, yellow, greenish pus was obtained from the right pleural cavity, July 16th, 1898, and July 17th he aspirated below and to the outer side of the other puncture, removing 33 ounces of thick, greenish-yellow pus. July 18th, aspirated and obtained 55 ounces; July 19th, aspirated and obtained same amount; July 20th, aspirated and removed 38 ounces of pus; July 27th, aspirated and removed 22 ounces of pus. A total of 241 ounces was aspirated in twelve days. In the operation portions of the ribs on the right side, from the second to the ninth inclusive, were removed, amounting to a total of 36 inches of rib. Excellent result!

Dr. Samuel Lloyd, of New York, contributed a paper on "Cancer of the Breast."

He is free to confess that he has for many years declined to accept the embryonic cell theory as a satisfactory explanation for the cause of this disease, and it has been to his mind a strong argument in favor of some infectious element that cancer should be on the increase. Locality seems to have a decided influence in the production of the disease, and some years ago he collected considerable data bearing on this point in an editorial in the *Annals of Surgery*, and there has been recently an article on this same subject embodying the results of later investigations in several numbers of the *Centralblatt für Chirurgie.* Perhaps more startling than this clinical experience, however, is the almost simultaneous announcement from the New York State Laboratory, from Dr. Armand Ruffer's Laboratory, and from Dr. Bra, of Paris, that they have discovered certain bodies in cancer cells which seem to be distinctive of the disease. More surprising still are the results of Bra, for he has advanced still further than the other observers. In six personal observations, which increases the total to twenty-two cases of cancer of the uterus, mamma, ovary or rectum, or with epithelioma of the tongue or cervix, in which these parasites have been noted in the blood, he has succeeded, in injecting animals with cultures of this fungus, derived from human cancer, in producing tumors with the typical structure of fibro-sarcoma and carcinoma.

Accepting these statements, together with the clinical data already published, surgeons find the dawn of a new era in the management of this disease.

From the time he began to operate he performed the Volkmann operation, and he has one patient, operated upon in 1885, who lived seven years without recurrence; she died of pneumonia in 1892. After Heidenhain's paper he made it a habit to remove the whole of the pectoralis major, except the clavicular portion, and following the papers by Halstead and Meyer, he extended this to complete removal of the pectoralis major and the other structures advocated by those operators. He has one patient, the only one he can trace, who is now in the tenth year of freedom from recurrence following the incomplete removal of the pectoralis major. Within the past few years the importance of the complete or radical operation has been forced upon him more strongly than ever. It is frequently the case in his experience that the axillary lymphatics do not seem to be involved, and yet after the pectoralis minor has been cut through and the infraclavicular glands and fat exposed, it is evident that they were infiltrated. In these cases any operation short of a careful exposure of the whole infraclavicular region would have left definite areas of infection, and this probably accounts for the number of cases where it is observed that the recurrence came just under the clavicle.

Every tumor of the breast should be removed as soon as possible, and time should not be wasted in vain attempts to absorb it. If there is reason to believe that the growth is benign, it may be cut into first, and then the operator may determine whether it is suspicious or not. But every suspicious tumor of the breast should lead to a complete extirpation of the breast at once. Age is no protection from cancer, and we should not forget that it may appear in early as well as in late life. Dr. Lloyd has operated for cancer, and the pathological report has sustained the diagnosis, in a woman of twenty-six, and in several between that age and thirty.

Dr. James B. Baird of Atlanta, read a paper in which he dwelt upon "The Rights and the Wrongs of the Medical Witness in Courts of Justice."

It seems to him that no argument beyond the bare statement of the facts is necessary to sustain the view that the medical profession and the people suffer in consequence of inadequate laws upon these points. If the laws of the State are inadequate or defective, such inefficiency and defect must constitute an evil; and if the citizen in consequence of such imperfection does suffer, there must be a wrong. So if the evil and the wrong really exist, appropriate legislation judicially applied is the only and the proper remedy. Legislation which makes confidential communications to the physician privileged communications, and which provides, in the discretion of the court, just compensation for expert medical services, will correct the evil and right the wrong, and toward the procurement of such legislation in the interest of the medical profession and of the people of this commonwealth, the united efforts of the Association should be diligently addressed.

SECOND DAY—AFTERNOON SESSION.

Dr. Hunter P. Cooper of Atlanta, reported a case of gunshot wound of the abdomen, and exhibited the patient.

Dr. J. L. Hiers of Savannah, read a paper on "Nasal Obstruction and its Influence."

Dr. Joseph Price of Philadelphia, contributed a paper by invitation on "An Analysis of Appendicitis Operative Methods," which was followed by a paper on "Appendicitis from the Standpoint of a Country Doctor," by Dr. A. C. Davidson of Sharon.

Dr. James M. Crawford of Atlanta, read a paper on "Mouth-Breathing," and reported the following case:

M. N., aged seven; mouth-breather for last four years; also aural catarrh. Hears watch three inches in either ear. Has been under the care of a specialist for at least three and a half years who used sprays, inflating the middle ears, etc. November 1, 1895, he operated upon her for adenoids, removing considerable soft tissue with his finger-nail. Hearing was soon restored to normal, and now, after a lapse of more than three years, there has been no return of the deafness, and the nasal breathing has been fully restored.

Dr. Ross P. Cox of Rome, followed with a paper on "Laryngeal Diphtheria Cases with Intubation."

Dr. A. A. Davidson of Augusta, read a paper on "Mumps and Metastasis."

Dr. George H. Noble of Atlanta, demonstrated by means of charts and diagrams an improved method of performing perineorrhaphy.

Dr. J. D. Herrman of Eastman, read a paper on "Cold in the Treatment of Pneumonia."

Cold has been applied in the treatment of this disease by Germans for a long time, and chiefly in the hospitals of this country. The cold must be applied continuously and persistently. How is it applied? The ice must be broken in pieces small enough to be put into a small mouth rubber bag; and the ice-bag is wrapped in a towel, is placed against the affected area, and is changed if the disease extends. The length of time that ice should remain is governed entirely by the wave of the temperature; if this falls to or near the normal point, the ice should be removed, and again be applied should there be a tendency to elevation of temperature. The ice not only reduces the temperature, but checks the further spread of pulmonary congestion. He has reason to believe that it produces resolution in areas which are in the stage of exudation. He considers no age a bar to its use if the temperature warrants it. Next to ice, strychnia and digitalis are probably of the greatest importance. So far as he has observed, ice has no injurious effect on patients with pneumonia. He has had good results from this mode of treatment for the past few years.

THIRD DAY——MORNING SESSION.

This session was largely taken up with a symposium on "Anesthesia."

Dr. Louis H. Jones of Atlanta, read a paper entitled "Crawford W. Long and his Discovery."

He stated that Dr. Long was born in Danielsville, Madison county, Ga., November 1, 1815. When quite young he entered Franklin College, now the University of Georgia, and graduated at the age of nineteen, taking the second honor in his class. He studied medicine and graduated at Jefferson Medical College in 1839.

On March 30, 1842, Dr. Long administered ether to a young white man, named James M. Venable, for the purpose of excising a small tumor located on the back of his neck. The ether was given until the patient became insensible, and was continued during the operation, the patient showing no sign of pain, and after regaining consciousness refused to believe that the tumor had been removed until it was shown to him. A month or two later a second tumor was removed from the neck of the same patient, ether being again administered but discontinued after the patient became anesthetized and before the operation began. On this occasion Venable stated that he felt slight pain as the last cut was being made.

The essayist read a copy from Dr. Long's journal of charges against James M. Venable for medical services, which we give below:

James Venable to Dr. C. W. Long, Dr.

| | |
|---|---|
| 1842. | |
| Jan. 28—Sulphuric ether | $ 25 |
| March 30—Sulphuric ether and exsecting tumor | 2 00 |
| May 13—Sulphuric ether | 25 |
| June 6—Exsecting tumor | 2 00 |
| Total | $4 50 |

Georgia, Jackson county.

In an article in the *Virginia Medical Monthly*, May, 1878, Dr. Marion Sims writes as follows:

" . . . Sixth. That Long was the first man to intentionally produce anesthesia for surgical operations, and that this was done with sulphuric ether in 1842. Seventh. That Long did not by accident hit upon it, but that he reasoned it out in a philosophical and logical manner. Eighth. That Wells, without any knowledge of Long's labors, demonstrated in the same philosophical way the great principle of anesthesia by the use of nitrous oxide gas, in 1844. Ninth. That Morton intended to follow Wells in using the gas as an anesthetic in dentistry, and for this purpose asked Wells to show him how to make the gas in 1846. Tenth. That Wells referred Morton to Jackson for this purpose, as Jackson was known to be a scientific man and an able chemist. Eleventh. That Morton called on Jackson for information on the subject, and that Jackson told Morton to use sulphuric ether instead of nitrous oxide gas, as it was known to possess the same properties, was as safe and easier to get. Twelfth. That Morton, acting on Jackson's offhand suggestion, used ether successfully in the extraction of teeth (1846). Thirteenth. That Warren, Heyward and Bigelow performed important surgical operations in the Massachusetts General Hospital, in October, 1846, on patients etherized by Morton, and that this introduced and popularized the practice throughout the world. . . . To each is due a certain measure of credit, but no man can claim this great honor exclusively. The names of Long, Wells, Morton and Jackson will doubtless be associated as colaborers in the great work, and to these must be added the immortal name of Sir James Y. Simpson, who introduced chloroform and enlarged the domain of anesthesia."

Dr. J. B. Morgan, of Augusta, followed with a paper on "Physiology of Ether Anesthesia."

The author knows of no case of death from ether during the primary stage of anesthesia, but if the vapor is inhaled in a too concentrated form, there is usually, in the beginning, a momentary arrest of respiration and a decided sense of suffocation, evidently the result of the irritant action of the vapor upon the mucous membranes of the upper air passages.

In regard to the physiology of ether anesthesia, two conclusions are well established:

1. That the vapor of ether is dissolved in the blood of the pulmonary capillaries; it is then carried to the left side of the heart, and from there finds its way through the circulation to every part of the system.

2. That ether affects the nerve centers; first the cerebrum, then the sensory centers of the cord, next the motor centers of the cord, then the sensory centers of the medulla oblongata, and finally the motor centers of the medulla.

Dr. William H. Elliott, of Savannah, read a paper on "Ether *vs.* Chloroform."

Chloroform is considered safe in children and in obstetric practice. It is contraindicated in large, weak, or fatty heart, and it is said to be especially dangerous where orthopnea exists. Chloroform must be promptly discontinued in any case where alarming symptoms attend its use.

The chief danger in the administration of chloroform is from paralysis of the heart, and the risk is greater at the beginning of the inhalation. The more concentrated the vapor, the greater the danger.

In ether, on the other hand, the chief dangers are not during the inhalation, but after, and come in the form of respiratory or genito-urinary complications, especially suppression, and occasionally there is unaccountable collapse. Should a fatal result ensue, it would be more likely to be charged to disease than to the anesthetic.

To sum up: He is satisfied that chloroform is the pleasanter, quicker and more satisfactory anesthetic. He thinks most of the dangers can be avoided by the skill and care of an administrator who realizes that anything that can put to sleep, can kill. To the careless or inexperienced he would recommend ether. Some years ago, at a meeting of the National Association of Railway Surgeons, a test vote was taken on the subject of anesthetics. The question came up by chance, and the attendance at the moment was not large. The vote resulted, 16 in favor of ether, and 100 in favor of chloroform. "Let every man be fully persuaded in his own mind." "Happy is he that condemneth not himself in that thing which he alloweth."

Dr. Samuel C. Benedict, of Athens, followed with a contribution on "The Administration of Ether and How to Meet its Dangers."

The author reviewed the methods of its administration, and later discussed the proper way to meet the dangers to the patient arising from such administration, so as to guide him to a safe awakening.

Dr. James P. Tuttle, of New York, delivered an address by invitation on "The Technique of Rectal Examination."

Among the symptoms which suggest rectal examination he called attention to the following: (1) Vague pains or aching about the pelvis, sacrum, or coccygeal region, and shooting down the left leg. (2) Flatulence, with indigestion, loss of appetite, and irregularity or constipation of the bowels. (3) Tendency to diarrhea, especially in the morning. (4) Sense of constriction or weight about the pelvis. This is especially important in males. (5) Spasmodic or periodic dysuria, without adequate cause in the genito-urinary apparatus. (6) The presence of mucus, pus, shreds or blood in the dejections. (7) Irregular menstruation or dysmenorrhea, especially in young women and schoolgirls. (8) Picking of the nose, scratching of the abdomen, restlessness at night, and constipation in young children.

Before the actual examination of the rectum is begun, certain subjective symptoms should be inquired into and their suggestions as to the probable cause be borne constantly in mind. Among these may be mentioned:

1. The habitual state of the bowels—whether regular, constipated or diarrheal. If constipated, is the stool, when it appears, soft and consistent, of normal shape, tape-like, or hard and ball-like? Is it clean or covered with mucous or tinged with blood? If diarrheal, is it watery, free and painless, or scanty mucous, and attended with pain and subsequent exhaustion?

2. Pain—when does it occur, at, before or after stool; how long does it last; what is its nature, acute, cutting, burning, or a dull ache? Where is it experienced, at the anus, the sacral region, about the pelvis, in the inguinal region, or shooting down the leg; or is it, as often happens, in the uterus, neck of bladder, or urethra?

3. Itching and spasmodic twitching of the sphincter.

4. Discharge—what is its character—bloody, mucous, watery, purulent or fetid? Is it alone or mixed with fecal matter? When does it occur, at, before, after or independent of the stool?

5. Protrusion—when does it occur; is it brought on by long straining or upon slight exertion; is it pronounced; does it disappear spontaneously or is it difficult to restore; is it hard or soft, smooth and general, or localized and nodular? If down at the time of the examination, the direction of the rugæ, whether circular or running up and down, should be carefully observed, as in these we have the pathognomonic distinction between complete and partial procidentia.

6. What are the habits and history of the patient? Has there been much use of enemas; is he in the habit of sitting long at the shrine of Cloacus with his pipe and paper as companions; is there an unsatisfied sensation after stool, a feeling of something more to come away; is he the victim of pederasty; has he a history of gonorrheal or syphilitic infection, or has he hereditary tendency to tuberculosis or malignant disease?

Dr. Tuttle then described in detail methods of examination and treatment of various diseases of the rectum, which, if systematically followed out by all physicians, intractable and incurable diseases of the rectum would soon become things of history instead of nightmares to the profession.

Dr. Alex. W. Stirling, of Atlanta, read a paper entitled "Albuminuria, Including Original Investigations and its Relation to Diseases of the Eye."

When dealing with the relationship of albuminuria to eye diseases, the oculist considers first and chiefly albuminuric retinitis. It would be a great oversight to omit entirely other affections which are equally closely related to it and are dependent upon the same cause. They may very well be divided into those which depend upon lesions of the visual apparatus—first, outside the brain, among which is albuminuric retinitis itself, and, second, within the brain. Among the former are edema and hemorrhage in the lids and conjunctiva; hemorrhage into the optic nerve and into the orbit. Embolism or thrombosis of the central vessels of the retina is probably less uncommon than has been supposed. Iritis and cataract are probably little if any more common in albuminurics than in the general population.

Among intracranial lesions, hemorrhage into the nuclei or trunks of the nerves supplying them has been said to produce temporary paralysis of some of the external muscles of the eye. Uremic amblyopia is more frequently met with. It is probably the result of extreme cerebral anemia due to vascular contraction, caused by the irritation of poisonous matters in the circulation. The resulting blindness usually affects both eyes, and may be incomplete and transient, or absolute, vision returning only after some hours' duration, though it may be delayed for three or four days. The pupils generally react to light, which indicates the cortical origin of the attack, but occasionally they do not, and then the nerve or optic ganglia are involved. In these latter cases the optic disc is likely to be found swollen. Where recovery of vision does not fully take place, there must be some organic lesion, and *post-mortem* examinations have shown that apparently functional cases have sometimes had their origin in small hemorrhages. It should also be noted that central amblyopia is not infrequently accompanied by other nerve symptoms, such as hemiplegia.

Among other papers read at this meeting were the following: "Chronic Diarrhea," by Dr. T. M. Greenwood, of Mineral Bluff; "Some Thoughts on Malpositions and Malpresentations," by Dr. Edwin H. Sims, of Columbus; "Asphyxia Neonatorum; Prognosis and Treatment," by Dr. C. H. Richardson of Montezuma; "Prevention and Treatment of Pelvic Inflammatory Diseases in the Female by the General Practitioner," by Dr. R. R. Kime of Atlanta.

The following officers were elected for the year: President, Dr. F. W. McRae of Atlanta; Vice-Presidents, Drs. St. J. B. Graham of Savannah, and H. B. McMaster of Waynesboro.

Atlanta was selected as the place for holding the next meeting, and the time the third Wednesday in April, 1900.

